# Protein alignment algorithms with an efficient backtracking routine on multiple GPUs

**DOI:** 10.1186/1471-2105-12-181

**Published:** 2011-05-20

**Authors:** Jacek Blazewicz, Wojciech Frohmberg, Michal Kierzynka, Erwin Pesch, Pawel Wojciechowski

**Affiliations:** 1 Poznań University of Technology, Poznań, Poland; 2 Institute of Bioorganic Chemistry PAS, Poznań, Poland; 3 University of Siegen, Siegen, Germany; 4 Poznań Supercomputing and Networking Center, Poznań, Poland

## Abstract

**Background:**

Pairwise sequence alignment methods are widely used in biological research. The increasing number of sequences is perceived as one of the upcoming challenges for sequence alignment methods in the nearest future. To overcome this challenge several GPU (Graphics Processing Unit) computing approaches have been proposed lately. These solutions show a great potential of a GPU platform but in most cases address the problem of sequence database scanning and computing only the alignment score whereas the alignment itself is omitted. Thus, the need arose to implement the global and semiglobal Needleman-Wunsch, and Smith-Waterman algorithms with a backtracking procedure which is needed to construct the alignment.

**Results:**

In this paper we present the solution that performs the alignment of every given sequence pair, which is a required step for progressive multiple sequence alignment methods, as well as for DNA recognition at the DNA assembly stage. Performed tests show that the implementation, with performance up to 6.3 GCUPS on a single GPU for affine gap penalties, is very efficient in comparison to other CPU and GPU-based solutions. Moreover, multiple GPUs support with load balancing makes the application very scalable.

**Conclusions:**

The article shows that the backtracking procedure of the sequence alignment algorithms may be designed to fit in with the GPU architecture. Therefore, our algorithm, apart from scores, is able to compute pairwise alignments. This opens a wide range of new possibilities, allowing other methods from the area of molecular biology to take advantage of the new computational architecture. Performed tests show that the efficiency of the implementation is excellent. Moreover, the speed of our GPU-based algorithms can be almost linearly increased when using more than one graphics card.

## Background

The most important and the most frequently used algorithms in computational biology are probably the Needleman-Wunsch [1] and the Smith-Waterman [2] algorithms for global and local pairwise alignments of DNA (and protein) sequences, respectively. These algorithms are based on dynamic programming. As a result, one gets an optimal alignment, but the approach requires a lot of time and memory. The problem becomes more serious when pairwise alignments have to be computed for a set of thousands of sequences (a common case at the assembly stage of DNA recognition [3-5]). A natural extension of the pairwise alignment is a multiple sequence alignment (MSA) problem, which is much more complex. Theoretically, the MSA problem can be also solved by dynamic programming, but it was proved that for a Sum-of-Pairs score this problem is NP-hard [6]. Thus, heuristic approaches are frequently used (see review [7]). The most common ones, based on the so called progressive algorithm, require the alignment of every input sequence pair. Sometimes, such pairwise alignments are performed with highly specialized methods like in case of [8,9], but often it is the Needleman-Wunsch or Smith-Waterman algorithm [10,11] resulting in time-consuming methods. Hence, the increasing number of sequences is perceived as one of the upcoming challenges for the MSA problem in the nearest future [12].

Recently, modern graphics processing units (GPUs) have been widely exploited for solving many bioinformatic problems. An example may be the problem of scanning databases for sequences similar to a given query sequence. A few efficient implementations addressing this problem have been developed (see [13-16]). However, it should be stressed that scanning a database is considerably different from aligning every possible pair of sequences from a given input set. Both problems, seemingly the same, vary in many aspects, especially in case of low-level GPU optimizations. Moreover, it is worth noting that all the methods mentioned above compute only the alignment score, not the alignment itself. Yet, many real-life applications require also the alignment to be computed. One of the known approaches, by Khajeh-Saeed A. et al. [17], partially solves this problem but the application has been designed for a very specific benchmark. Additionally, the method adopted for backtracking procedure is not clear and not very efficient either. Hence, the software is not applicable in practice, e.g. to the MSA or the DNA assembly problem. However, the idea presented by Liu Y. et al. [18] seems to be a quite successful approach to the former of these two problems. The proposed solution uses the Myers-Miller algorithm [19] to compute the alignment. The main advantage of this algorithm is the possibility of aligning very long sequences as the backtracking procedure works in linear space. The main drawback, on the other hand, is the necessity of conducting additional computations - the backtracking routine has quadratic computational complexity here. But yet, many practical applications require dealing with a large number of short sequences, e.g. [20]. In these problems a special emphasis should be put on efficient processing without any redundant or repeated computations and not necessarily on saving memory.

The main goal of this work derives from the discussion above. It is a construction of GPU-based dynamic programing algorithms for pairwise alignment. One difference between our approach and the previous ones is that we have optimized the algorithm for aligning every sequence with each other from a given input set. The second difference is that our method, unlike others, performs the backtracking procedure in linear time. Although special data structures are used here, no redundant computations are needed. In contrast to the Myers-Miller algorithm, it was designed for the GPU architecture. Moreover, the three basic pairwise alignment algorithms, i.e. local, global and semiglobal, differ only in details, so all of them have been implemented. As a result we got a valuable tool for multi-sequence pairwise alignments which is fast and can be run on a common personal computer equipped with NVIDIA GPU (G80, GT200 or Fermi). Extensive computational tests show its advantage over CPU-based solutions like the Emboss package or the highly optimized Farrar's implementation. Moreover, our task manager is able to use more than one GPU. Performed tests show that the multi-GPU support influences the execution time considerably.

### GPGPU and the CUDA programming model

There are a few substantial differences between CPU and GPU architectures that make a GPU a more powerful tool for some applications. The same differences cause some difficulties in programming of graphics cards. Firstly, GPUs have many more cores, which are the main computational units, e.g. NVIDIA GeForce 280 has 240 cores. Secondly, there is much less cache memory available on the GPU. Moreover, the cache memory on the graphics card is not managed automatically, but by a programmer.

Such an architecture gives opportunities to utilize the hardware more efficiently. On the other hand, writing parallel algorithms on GPU is more time-consuming, because it requires in-depth knowledge and understanding of the hardware. As a result the algorithm can be much faster than its CPU version. Although there are a few GPGPU (general-purpose computing on graphics processing units) technologies like ATI Stream [21] or OpenCL [22] on the market, one of them - CUDA [23], is a bit more established than others. Our implementation of alignment algorithms was done using this technology. The CUDA environment is an extension of C/C++ programming languages which enables programmers to access the resources of the GPU.

To understand the essentials of CUDA, one has to be aware of different types of available memory. The main differences between these memory types have been shown in Table 1. The proper usage of memory is the key to good performance. However, not only the type of memory used is important, but also their correct usage. Different kinds of memory have different access patterns. It means that for instance, the order of reading/writing data can be also crucial [24]. Because RAM (also called the main or global memory) is much slower than the memory on the chip, most of the CUDA programs follow this simple rule: fetched data from the global memory is processed locally as much as possible, using registers, shared memory and caches, then the results are written back to the global memory. In this way one can limit expensive data transfers from or to the global memory.

**Table 1:** Differences between memory types in CUDA

Memory type	Located on chip	Cached	Access	Scope
Registers	yes	n/a	R/W	Thread

Local	no	no/yes*	R/W	Thread

Shared	yes	n/a	R/W	Block

Global	no	no/yes*	R/W	Program

Constant	no	yes	R	Program

Texture	no	yes	R	Program

Differences between memory types in CUDA (n/a stands for „not applicable”, letter R for „read” and letter W for „write”, * - caching depends on compute capability). For more information see CUDA Best Practices Guide [24].

Another significant property of CUDA-enabled graphics cards is that the GPU consists of many multiprocessors and each multiprocessor has a number of cores working as one or more SIMT (single instruction multiple thread) units. One such unit is able to execute one and only one instruction at the same time, but in many threads and on various parts of data. As a result, during the process of designing an algorithm, one must take this into consideration.

Being conscious of the architecture described briefly above, one can design and implement alignment algorithms efficiently.

### Algorithms for pairwise sequence alignment

There are three basic algorithms for performing pairwise sequence alignment: Needleman-Wunsch [1] for computing global alignment, its modification for semiglobal alignment and Smith-Waterman [2] for computing local alignment. All these algorithms are based on the idea of dynamic programming and to some extent work analogically. Taking into consideration Gotoh's enhancement [25], the algorithms are described briefly below.

Let us define:

• *A *- a set of characters of nucleic acids or proteins,

• *s_i _*- the *i*-th sequence,

• *s_i_*(*k*) ∈ *A *- *k*-th character of the *i*-th sequence,

• *SM *- substitution matrix,

• *SM*(*c_i _*∈ *A*, *c_j _*∈ *A*) - substitution score for *c_i _*and *c_j _*pair,

• *G_open _*- penalty for opening a gap,

• *G_ext _*- penalty for extending a gap,

• *H *- a matrix with partial alignment scores,

• *E *- a matrix with partial alignment scores indicating vertical gap continuation,

• *F *- a matrix with partial alignment scores indicating horizontal gap continuation,

#### Needleman-Wunsch algorithm

To compute the alignment of two sequences, the algorithm (called later NW algorithm) has to fill the matrix *H *according to the similarity function. The similarity function determines a score of substitution between two residues. This relation is given in a substitution matrix, like one from BLOSUM [26] or PAM [27] families. The matrix *H *also takes gap penalties into account, described by *G_open _*and *G_ext_*. The size of the matrix *H *is (*n *+ 1) × (*m *+ 1), where *n *is the number of residues in the first sequence *s*_1 _and *m *- in the second sequence *s*_2_.

The matrix *H *is filled using the following formulae:(1)(2)(3)

where *i *= 1...*n *and *j *= 1...*m*.

The first row and the first column are filled according to the following formulae:(4)(5)

Moreover, the *E *and *F *matrices are initialized by putting -∞ value into the first row and column. The result for this part of the algorithm is the value of similarity, so called score. Let us denote the coordinates of the cell with the similarity score by (i*, j*). In case of the NW algorithm, this value can be found in the *H*(*n*, *m*) cell of the matrix *H*.

The goal of the second stage - backtracking, is to retrieve the final alignment of two sequences. The idea of backtracking is that the algorithm performs backward moves starting from the (i*, j*) cell in the matrix *H *until it reaches the (0, 0) cell. Every time when the algorithm moves to the upper cell, a gap character is inserted into the sequence *s*_1 _in the final alignment. If the algorithm moves left, a gap is added analogically to the sequence *s*_2_, and finally the diagonal move means that the corresponding residues are aligned. The backtracking procedure is deeply analyzed in Section "The idea of backtracking procedure and GPU limitations".

#### The semiglobal pairwise alignment

A semiglobal version of dynamic programming for pairwise alignment differs from the previous one in three points. The first one is the way how the matrix *H *is initialized. For semiglobal alignment the formulae (4) and (5) should be replaced respectively by:(6)(7)

The second difference concerns the coordinates of the cell where the similarity score can be found in the matrix *H*. For semiglobal alignment this cell is the one with the highest value from the last row or column of the matrix *H*.

The last difference involves the stop criterion for the backtracking procedure. In this case backtracking is finished when the cell (*k*, 0) or (0, *l*) is reached, where *k *= 0, ..., *n *and *l *= 0, ..., *m*.

#### Smith-Waterman algorithm for the local pairwise alignment

The Smith-Waterman algorithm (called later SW algorithm) also differs from the Needleman-Wunsch algorithm in three points. The first one is again the way of initializing the matrix *H*. The initializing values should be the same as in the semiglobal version of the algorithm.

The second difference concerns the formulae describing the process of filling the matrix *H*. The formula (1) should be replaced by the following one:(8)

where *i *= 1...*n *and *j *= 1...*m*.

The next difference covers the coordinates of the cell with the final score for the local alignment. In this case the (i*, j*) cell is the one with the highest value within the entire matrix *H*.

The last difference concerns the stop criterion of the backtracking procedure. The Smith-Waterman algorithm is finished when a cell with zero value is reached.

## Implementation

### The idea of backtracking procedure and GPU limitations

To obtain the alignment efficiently four boolean matrices have been defined in our approach, each of size (*n *+ 1) × (*m *+ 1). The purpose of these matrices is to indicate the proper direction of backward moves for the algorithm being at a certain position during the process of backtracking. Although their memory usage is quadratic, the advantage is that they enable to perform the backtracking procedure in a linear time, in contrast to the Mayers and Miller's idea.

The backtracking matrices are defined as follows:

• *C^up ^*- indicates whether the algorithm should continue moving up,

• *C^left ^*- indicates whether the algorithm should continue moving left,

• *B^up ^*- indicates whether the algorithm should move up, if it does not continue its way up or left,

• *B^left ^*- indicates whether the algorithm should move left, if it does not continue its way up or left.

Two special cases should be stressed:

• if *C^up ^*= *false*, *C^left ^*= *false*, *B^up ^*= *true *and *B^left ^*= *true *then the algorithm should move to the diagonal cell in the up left direction,

• if *C^up^*, *C^left^*, *B^up ^*and *B^left ^*have logical value *false *then the backtracking procedure is finished.

In the case of global and semiglobal alignment algorithms, the matrices are filled according to the following formulae:(9)(10)(11)(12)

The additional condition of *H_i,j _*≠ *E_i,j _*in the formula (12), as compared to the formula (11), prevents the algorithm from an ambiguous situation, when both directions, up and left, are equally good. In this case, to avoid non-deterministic behavior, the algorithm should prefer only one, predefined direction.

For the local alignment algorithm, the *C^up ^*and *C^left ^*matrices are filled according to formulae (9) and (10), respectively. However, the *B^up ^*and *B^left ^*matrices are filled using the following formulae:(13)(14)

An important issue, one should take into consideration, is that during the process of filling the matrix *H *any cell value can be computed only if the values of the left, above and diagonal cells are known. It means that only these cells that are on the same anti-diagonal can be processed simultaneously. As a result, there is not much to parallelize (in the context of massively parallel GPU architecture) in a single run of NW or SW algorithm. However, progressive multiple sequence alignment algorithms require aligning of many sequences (every sequence with each other). Our idea was to design an algorithm for efficient execution of many pairwise alignment instances running concurrently. To utilize the GPU resources properly one has to load it with a sufficient amount of work. To fulfill this requirement at least 80 × 80 NW/SW instances should be computed concurrently (this number will be explained in Section "Implementation of the algorithms"). The problem to overcome was that the amount of available RAM on graphics cards was, for this purpose, relatively small (e.g. the GeForce GTX 280 usually has 1 GB of RAM). In fact, the *H*, *E *and *F *matrices do not have to be kept entirely in RAM (see Section "Implementation of the algorithms"). However, the backtracking tables (*C^up^*, *C^left^*, *B^up^*, *B^left^*) must be kept in the global memory. Hence, they would take a lot of memory space if they were held in normal C/C++ boolean arrays, e.g. for sequences with lengths of 500 residues one would need 80 × 80 × 500^2 ^× 4 bytes i.e. 6103.5 MB only for backtracking arrays. Thus, a special emphasis has been put to make this figure smaller, so that the algorithm can be run on any CUDA-capable device.

### Implementation of the algorithms

All the algorithms in our implementation, namely the NW algorithm, its semiglobal version and the SW algorithm, have a few input parameters such as a substitution matrix, *G_open _*and *G_ext _*values, a file in *fasta *format with sequences to be aligned, etc. When the algorithm is launched, it performs an alignment of every given sequence with each other. The result of the algorithm consists of the score and the alignment for each pair of sequences.

Let *S *be the set of input sequences. The total number *N *of sequences' pairs to be aligned is given by the following formula:(15)

**Figure 1:**
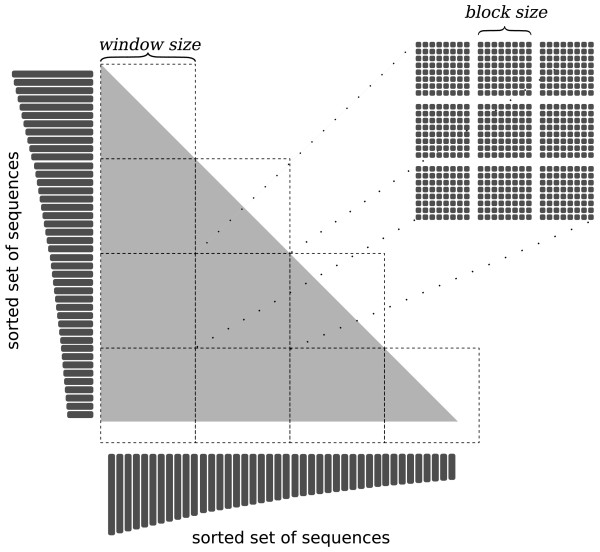
**Division of the problem**. Division of the problem into subproblems called *windows *and *blocks*. The input sequences are sorted from the longest to the shortest one.

Because the problem of aligning many sequences simultaneously is a memory-consuming task, it has been split into subproblems. The whole matrix of tasks, where a single task is a pairwise alignment, was divided into smaller matrices, called *windows *(see Figure 1). The size of each *window*, denoted by *window size*, is a trade-off between the amount of global memory required and the number of tasks running concurrently. Obviously, the global memory is limited and the number of tasks running concurrently is directly connected with the efficiency. Performed tests showed that a good value for the *window size *parameter is 80 - this number can vary depending on the hardware. The vast majority of memory allocated by the algorithm is used for backtracking matrices. Therefore, one of the most significant problems was to store them properly. It is crucial to pack backtracking matrices into as small memory space as possible. To achieve this, any single cell of previously defined boolean matrices (*C^up^*, *C^left^*, *B^up^*, *B^left^*) is represented by one bit. Hence, in one 32-bit memory word, a total of 32 values can be stored. These enhancements, i.e. windowing and backtracking matrices with their binary representations, enable the algorithm to run on any CUDA-capable device.

One *window *can be considered as a *grid *consisting of constant-sized *blocks*, as shown in Figure 1. Any single *window *is executed entirely on one GPU. Many *windows*, though, can be executed on many different GPUs. The *block size *is set to 16, because of our low-level optimizations of the algorithm. It means that in one block there are 16 · 16 = 256 threads. Each thread is responsible for aligning one pair of sequences. Although the *window size *value must be divisible by the *block size*, the algorithm ensures that all input sequences will be aligned, despite of their number. Execution of a *block *is over if its last thread finishes. Hence, to fully utilize the hardware resources, the lengths of all of the sequences within a *block *should be similar. The same applies to any *window *and its *blocks*. Therefore, the input sequences are sorted from the longest to the shortest one in the preprocessing step. This enhancement improves the algorithm performance significantly.

**Figure 2:**
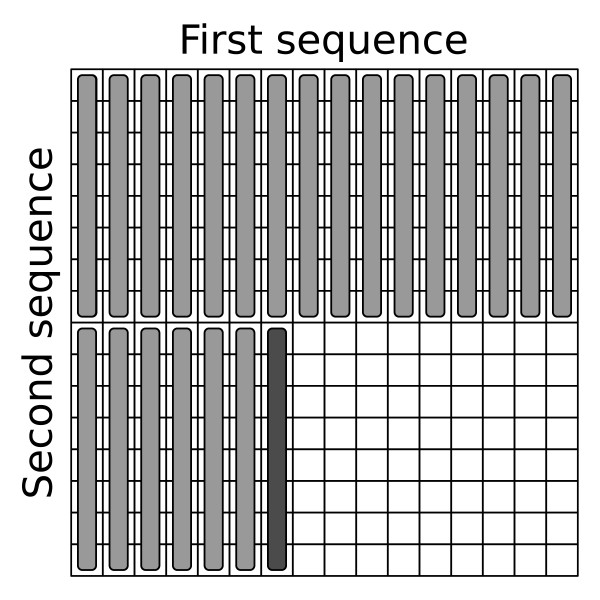
**Processing of the dynamic programming matrix**. Processing of the dynamic programming matrix. The cells are processed horizontally in a group of eight. The cells that have already been processed are marked as grey, cells that are currently processed are black and cells to be processed are white.

All alignment algorithms are divided into two main procedures, called *kernels*. The first *kernel *computes the alignment score and fills the backtracking matrices, the second one performs the backtracking stage. In the first *kernel *every thread fills its *H*, *E *and *F *matrices as well as its backtracking arrays *C^up^*, *C^left^*, *B^up^*, *B^left ^*horizontally. In each iteration a total of eight cells are computed, as shown in Figure 2. The global memory is accessed at the beginning of the iteration (when one element of the H and E matrices is read) and at the end (when the results, i.e. one element of the *H *and *E *matrices, and eight elements of backtracking arrays, are written back). A pair of *H *and *E *elements are stored together as one 32-bit word.

**Figure 3:**
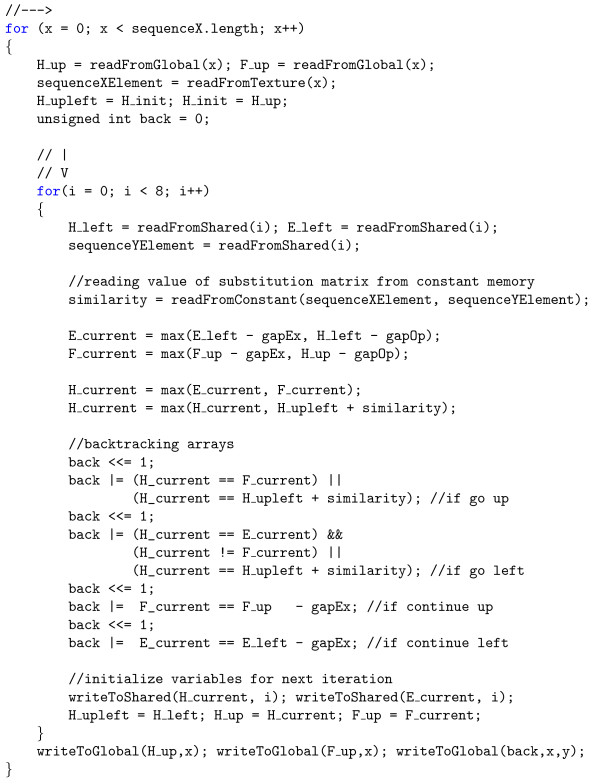
**Pseudocode of the first *kernel***. Pseudocode of the inner loops in the first *kernel*. The *H *matrix is filled in a way specific to the NW algorithm.

Also the elements of backtracking arrays are stored in a 32-bit word - eight elements in each of four matrices give totally 32 bits. Moreover, one can notice that the elements of the matrix *F *do not have to be transferred from/to the global memory, because they can be stored in the fast, shared memory. Although utilization of the shared memory greatly speeds up the algorithm, not all the solutions, e.g. Manavski et al. [13], leverage its potential. Additionally, in our implementation the elements of the substitution matrix are stored in the constant memory and the sequences are stored as a texture. As a result, to process eight elements of the dynamic programming matrix one 32-bit word is read from the slow, global memory and two 32-bit words are written back. Apart from this all the operations are performed using registers, shared memory, cached constant memory and textures. The pseudocode of the first *kernel *has been shown in Figure 3.

The idea of processing the dynamic programming matrix in vectors of eight elements in the first *kernel *is similar to the one proposed by Liu Y. et al. in CUDASW++ [15]. However, CUDASW++ kernel performs a database scan and, as such, takes advantage of storing the query sequence in the constant memory what results in significant performance boost. This idea was further exploited in CUDASW++2.0 [16] by using so called query profile. These improvements are not applicable for our solution in which there is no single query sequence that could be effectively shared across all the threads.

**Figure 4:**
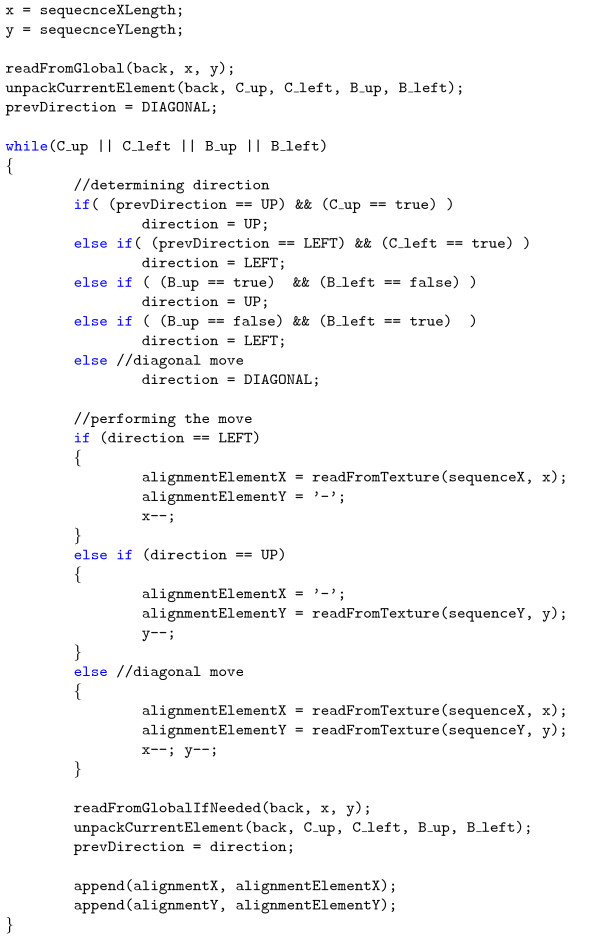
**Pseudocode of the second *kernel***. Pseudocode of the backtracking *kernel*.

The second stage of the algorithm - backtracking, is executed by the second *kernel*. Also in this case, one thread is responsible for processing of only one alignment. The *kernel *starts from the (i*, j*) cell, computed in the first stage, and performs the up, left or diagonal moves, depending on the backtracking matrices, until the stop condition is fulfilled. When the algorithm moves up, the elements of *C^up^*, *C^left^*, *B^up ^*and *B^left ^*matrices do not have to be read from the global memory, because in most cases, they are already in registers - one 32-bit word contains the information about eight elements of backtracking arrays. However, when the algorithm moves left or diagonal, one word is read from the global memory. This *kernel*, launched in *grids *of *blocks*, produces the final alignments of every sequence with each other. Its pseudocode has been shown in Figure 4. The second stage of the algorithm is very quick and usually comprises less than 1 percent of total runtime.

The advantage of using the backtracking arrays is that the backtracking stage can be performed very quickly in a linear time leading to very good solutions for short and medium-length sequences. However, its main drawback is quadratic memory complexity, discussed in Section "The idea of backtracking procedure and GPU limitations". Thus, the question is: what is the length of the longest sequences that can be processed by our program? Table 2 shows the maximum lengths of sequences, that can be aligned by the algorithm, depending on the amount of RAM available on the graphics card and the value of *window size *parameter. E.g. to utilize the resources of the GeForce GTX 280 with 1 GB of RAM properly, it is sufficient to set *window size *parameter to 80. It means that the input sequences, regardless of their number, can be as long as about 547 residues each. Processing of longer sequences is also possible, but the *window size *parameter should be decreased, e.g. the proper *window size *value for sequences with the length of 900 residues is 48. Although this change will have an influence on the overall performance of the algorithm, its speed may be still satisfactory. Taking this into consideration, we can conclude that the algorithm can process sequences with reasonable length. On the other hand, while aligning short sequences, one can try to increase the value of *window size*. This may improve the algorithm's performance.

**Table 2:** The maximum sequence length depending on the GPU RAM and *window size *parameter

*window size*	6 GB	4 GB	2 GB	1 GB	512 MB
16	7036	5721	3994	2751	1836

32	3518	2860	1996	1374	916

48	2345	1906	1330	915	609

64	1758	1429	997	685	455

**80**	**1406**	**1143**	**796**	**547**	**362**

96	1171	951	663	454	300

112	1003	815	567	388	255

128	877	712	495	338	221

144	779	633	439	299	194

160	701	569	394	268	172

176	637	516	358	242	155

192	583	473	327	220	139

208	538	436	301	202	126

224	499	404	278	186	115

240	465	376	259	172	105

Estimated length of the sequences that can be processed by the algorithm depending on the amount of the global memory (GPU RAM) and the *window size *parameter.

Bearing in mind, that nowadays many computer systems are equipped with more than one graphics card, we have designed and implemented a multi-GPU support. To ensure that all graphics cards used are equally loaded with work, regarding to their individual speeds, we have also implemented a task manager. Its role is to balance work among available GPUs. First, it sorts the tasks (here: *windows*) in descending order of their estimated complexity. Then, the tasks are assigned consecutively to any GPU that becomes idle. This type of scheduling, i.e. largest processing time first (LPT), although not optimal, ensures that the upper bound of execution time is equal to , where *t_opt _*is the optimal execution time and *m *- number of processing units [28,29]. In practice, applying LPT rule results in very good run times.

## Results

The main goal of this section is to compare the performance of the algorithm to other state-of-the-art approaches. However, before proceeding to the actual tests, the measure of *cell updates per second *(CUPS) should be well understood. The measure represents the average time needed to compute one cell in the matrix *H*, including the time of all side operations like computation of the values in the *E *and *F *matrices or performing the backtracking procedure. In practice, the number of computed cells in the matrix *H *is divided by the overall runtime of the algorithm. In our case it is:(16)

where *n *is the number of input sequences, *length_i _*is the length of the *i*-th sequence, *t *represents the time in seconds and the result is given in giga (10^9^) CUPS.

It should be stressed, however, that this measure underestimates the performance of the algorithms with a backtracking routine, because while the number of cells in the matrix *H *does not change, the time needed by backtracking is added.

The first implementation of the SW algorithm taking advantage of CUDA-capable GPUs has been developed by Manavski S. et al. [13]. The SW-CUDA algorithm running on two NVIDIA GeForce 8800GTX graphics cards achieves its peak performance of about 3.5 GCUPS. Another approach, developed by Ligowski L. et al. [14], with optimized shared memory usage was able to achieve up to 7.5 GCUPS using one and up to 14.5 GCUPS using both GPUs of the GeForce 9800 GX2. The CUDASW++ implementation by Liu Y. et al. [15] achieves a performance close to 10 GCUPS on a GeForce GTX 280 graphics card and up to 16 GCUPS on a dual-GPU GeForce GTX 295. This approach has been further explored by its authors resulting in optimized SIMT and partitioned vectorized algorithm CUDASW++ 2.0 [16] with an astonishing performance of up to 17 GCUPS on a GeForce GTX 280 and 30 GCUPS on a dual-GPU GeForce GTX 295. In the meantime also a couple of solutions addressing the Cell/BE [30-32] or FPGA [33,34] processors have been developed, all showing a great potential of new computing architectures. However, all implementations mentioned above solve a different problem - they perform a database scan, which is an easier problem to optimize for a couple of reasons, e.g. the query sequence may be kept all the time in fast on-the-chip memory. Moreover, all mentioned approaches concentrate on computing only the alignment score, not the alignment itself.

In search for an application to which our algorithm could be compared, we have come across the Khajeh-Saeed A. et al. solution [17]. This application, in one of its configurations, is able to perform the SW algorithm together with the backtracking stage and moreover apply this for any possible pair of sequences from a given input set. Thus, it is quite similar to our algorithm, but its performance is rather poor. Tests shown in the article indicate a performance of around 0.08 GCUPS for a GeForce GTX295 and about 0.17 GCUPS for four such graphics cards. Another application that performs the same computations is MSA-CUDA [18]. To be more precise, the first step of implemented ClustalW algorithm requires every input sequence to be aligned with each other. Unfortunately, the authors have not reported how fast this step is. Only the overall MSA times have been presented. Moreover, although the algorithm of Myers and Miller has been applied as a backtracking routine, sequences up to only around 860 residues have been tested in the article. Because the application is not available, we could not include it in our comparison. Since the number of state-of-the-art applications performing a backtracking procedure is very limited, we have decided to compare our algorithm to score-only implementations. In order to make them more similar to our approach, the score in a reference application should be calculated for any pair of sequences from a given input set. This assumption, however, ruins the performance of all GPU-based database scan solutions. In this case, they would have to be launched *n *- 1 times, where *n *is the number of input sequences, each time with decreased size of the database. Obviously, a good parallelism with a small database cannot be obtained for these algorithms. Hence, it would be unfair to include such tests in the paper. Instead, we decided to make use of a well-established algorithm of Farrar M. [35] which is a CPU-based, highly optimized database scan method. Yet, because a single run of the Smith-Waterman algorithm for two sequences is parallelized here, it can be easily modified into a version that computes a score for each pair of sequences without any loss of its performance. A detailed description of such a modification is provided is the next subsection.

### Comparison to the Farrar's implementation

In this test our algorithm is compared to the Farrar's implementation of the Smith-Waterman algorithm [35]. Farrar's approach utilizes the set of SSE2 instructions available in modern CPUs which makes the algorithm very efficient. The strength of the method does not rely on the great number of input sequences which can be processed simultaneously, but on SIMD operations performed within a single run of the SW algorithm. Therefore, the algorithm could be easily converted from scanning a database to computing scores for each pair of sequences from a given input set. All changes needed were made in the source code, so that the application does not have to be launched many times. Special tests were conducted to assure that the performance of the algorithm (in GCUPS) has not been affected.

For testing purposes we used the *Ensembl Databases *- *Release *55 [36], which contains genomic information regarding selected vertebrate species. All tests were performed on randomly selected subsets of sequences from the Homo Sapiens translation predictions.

In order to see if the performance of the algorithms depends on the length of the sequences, the input data was divided into six groups with different average lengths: 51, 154, 257, 459, 608 and 1103 amino acids. Moreover, for each group three sets with different number of sequences are considered to see if their number has any significant impact on the performance. The substitution matrix (BLOSUM50) as well as gap penalties (*G_open _*= 10, *G_ext _*= 2) were fixed the same for all tests.

The tests were run on the following hardware:

• CPU: 2 × Intel Xeon E5405, 2.0 GHz,

• GPU: NVIDIA Tesla S1070 with 16 GB of RAM,

• RAM: 16 GB,

• OS for our algorithm: 64-bit Linux,

• OS for Farrar's method: 64-bit Microsoft Windows 7.

Each algorithm was launched with each input data ten times. Table 3 presents the average execution times measured in seconds whereas Table 4 shows the performance in GCUPS. The standard deviation values *σ *have been omitted, because they do not give any significant information (in each case the value *σ *made up less than 1% of measurement). Additionally, our algorithm has been tested in one and four GPU configurations, respectively.

**Table 3:** Time comparison between our solution and the Farrars' implementation

avg. length	# of sequences	CPU, Farrar	1 GPU	4 GPUs
51	4000	24,113	8,064	2,070
	8000	95,156	31,111	7,855
	12000	210,806	69,083	17,439

154	2000	28,300	17,931	4,609
	4000	112,109	67,747	17,284
	6000	251,730	149,030	37,622

257	2000	61,182	49,226	12,535
	4000	242,756	186,436	47,305
	6000	543,656	410,255	103,278

459	2000	149,269	155,631	39,478
	4000	594,976	594,140	149,539
	6000	1339,538	1332,831	333,593

608	800	41,675	50,222	12,840
	1200	92,776	106,840	27,406
	1600	164,135	191,793	48,463

1103	800	123,572	164,780	41,946
	1200	278,194	359,065	89,899
	1600	495,624	628,847	158,699

Mean times (in seconds) for the Smith-Waterman algorithm applied to different sets of sequences. Average lengths of sequences as well as cardinality of sets are given. The Farrar's implementation computes only scores while our GPU-based implementation computes scores and alignments.

**Table 4:** Performance comparison between our solution and the Farrars' implementation

avg. length	# of sequences	CPU, Farrar	1 GPU	4 GPUs
51	4000	0,863	2,581	10,055
	8000	0,875	2,676	10,597
	12000	0,888	2,711	10,740

154	2000	1,677	2,647	10,296
	4000	1,693	2,801	10,980
	6000	1,696	2,865	11,349

257	2000	2,160	2,685	10,544
	4000	2,177	2,835	11,173
	6000	2,187	2,898	11,513

459	2000	2,824	2,709	10,679
	4000	2,834	2,837	11,274
	6000	2,831	2,846	11,370

608	800	2,842	2,358	9,224
	1200	2,871	2,493	9,720
	1600	2,885	2,469	9,770

1103	800	3,154	2,366	9,293
	1200	3,151	2,442	9,752
	1600	3,144	2,478	9,819

Mean values of GCUPS (Giga Cell Updates Per Second) for the Smith-Waterman algorithm applied on different sets of sequences. The GCUPS value is mainly used for score-only versions of the algorithm. Here, the performance of the GPU-based method is understated, because the value does not count in any additional operations (or cells) needed by the backtracking stage while the entire computational time is always considered.

The performance of the Farrar's algorithm grows significantly with increasing sequence length, reaching around 3.15 GCUPS for the sets with the longest sequences. In contrast, our algorithm with the performance of about 2.8 GCUPS seems to be insensitive to the sequence length. Its speed slightly decreases only for the groups with long sequences (608 and 1103). The reason behind this is that longer sequences require more global memory and thus the value of the *window size *parameter needs to be reduced. This corresponds directly to the number of tasks running in parallel. Hence the slowdown.

Farrar's solution, that has been used in this test, uses only one CPU core, but obviously we can expect a speedup close to linear if all CPU cores were used. However, our approach is also scalable - the execution times drop by a factor of nearly four when all four GPUs are used and the algorithm reaches up to 11.5 GCUPS. The number of input sequences does not affect the performance of Farrar's approach and it was high enough to have no influence on the performance of our algorithm. We can conclude that for sequences of average length (459) both implementations run comparably fast, but the GPU-based algorithm tends to be much faster when short sequences are processed. Moreover, it is worth noting that our algorithm additionally performs the backtracking step and computes the actual alignments of the sequences.

The speedup is much higher if the algorithm is compared to the one presented by Khajeh-Saeed A. et al. in [17]. Up to our knowledge this is the only GPU-based solution addressing the same problem where the performance is reported. Our approach is about 35 and 68 times faster for one and four graphics cards, respectively.

### Time comparison to the Emboss implementation

Apart from comparison to the state-of-the-art solutions, we decided to compare the algorithm to the implementation available in a popular package with bioinformatics tools - the *Emboss *[37]. The input sequences were also chosen from the *Ensembl Databases *- *Release *55 (see Section "Comparison to the Farrar's implementation"). We tested ten sets of sequences, each containing 2800 entries with lengths between 100 and 420 amino acids. The execution times of our NW and SW algorithms' implementations were compared with *needle *and *water *programs, which are available in the *Emboss *package. The *needle *program performs semiglobal alignments using the NW algorithm, the *water *program computes local alignments using the SW algorithm. These programs have been designed for aligning one sequence with a set of sequences. Instead of changing the source code, a special shell script was prepared that allows to align every sequence with each other. As a side effect, the programs from the *Emboss *package had to be launched 2800 times. In order to make the comparison reasonably fair their execution times have been reduced appropriately. We prepared a set of 2800 sequences with lengths equal to 1. Then, we measured the execution times of the programs from the *Emboss *package for this special set. The times were subtracted from the execution times of the test cases containing real sequences. It is worth noting that *needle *and *water *programs work using one CPU thread. Obviously, the NW and SW algorithms are deterministic - they always find the optimal solution. Therefore, there is no need to compare the quality of the results.

The tests were run on the following hardware:

• CPU: Intel Core 2 Quad Q8200, 2.33 GHz,

• GPU: NVIDIA GeForce GTX 280 with 1 GB of RAM,

• RAM: 8 GB,

• OS: 64-bit Linux.

**Figure 5:**
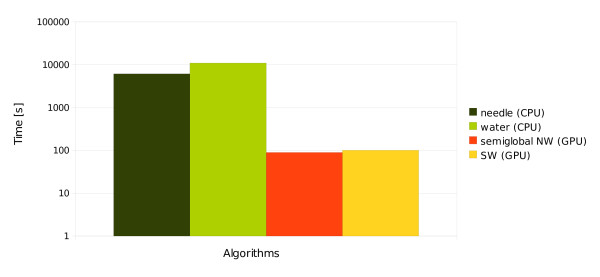
**Average time of aligning a set of 2800 sequences**. Average time of aligning a set of 2800 sequences. The algorithms marked as *needle *and *water *come from the *Emboss *package and ran on the CPU. The algorithms marked as *semiglobal NW *and *SW *were launched on a single GPU - GeForce GTX 280. The scale of the time axis is logarithmic.

Each algorithm was run ten times (once for each input set). Figure 5 shows the average execution times measured in seconds. The standard deviation values *σ *have been omitted, because they do not give any significant information (in each case the value *σ *comprised less than 1% of the measure).

The average times of computation for the *needle *and *water *programs were 6157 and 10957 seconds respectively (about 102 and 182 minutes), whereas the times for our implementation were as follows: 89.7 seconds for the NW and 100.8 seconds for the SW algorithm. Thus, the GPU implementation of the semiglobal version of NW was about 68 times faster than the CPU-based *needle*. In case of the SW algorithm the difference was even higher: the GPU version was about 108 times faster. To show this relationship properly, the scale of the time axis in Figure 5 is logarithmic.

### Multi-GPU test

The multi-GPU test was performed to see how the time of the computations depends on the number of graphics cards used. The sets of input sequences were the same as in the case of the test from Section "Comparison to the Farrar's implementation".

The tests were run on the following hardware:

• CPU: 2 × Intel Xeon E5405, 2.0 GHz,

• GPU: NVIDIA Tesla S1070 with 16 GB of RAM,

• RAM: 16 GB,

• OS: 64-bit Linux.

The Smith-Waterman algorithm was launched using one, two, three and four GPUs in turn. The algorithm was run ten times for each input set and the mean values of the computational times are shown in Table 5. To make the comparison easier, we added the columns with speedups. The execution times of the algorithm were nearly two times shorter for two graphics cards, nearly three and four times shorter when using three and four graphics cards, respectively. This shows that using more than one GPU one can gain almost linear speedup.

**Table 5:** Computational times of the SW algorithm depending on the number of GPUs used

avg.length	# of sequences	1 GPU		2 GPUs		3 GPUs		4 GPUs	
		
		time	speedup	time	speedup	time	speedup	time	speedup
51	4000	8,06	1,000	4,04	1,997	2,71	2,971	2,07	3,896
	8000	31,11	1,000	15,57	1,998	10,39	2,995	7,86	3,961
	12000	69,08	1,000	34,56	1,999	23,07	2,994	17,44	3,961

154	2000	17,93	1,000	9,05	1,982	6,10	2,939	4,61	3,890
	4000	67,75	1,000	34,21	1,981	22,93	2,955	17,28	3,920
	6000	149,03	1,000	74,66	1,996	50,14	2,973	37,62	3,961

257	2000	49,23	1,000	24,72	1,991	16,59	2,968	12,54	3,927
	4000	186,44	1,000	93,80	1,988	62,80	2,969	47,31	3,941
	6000	410,26	1,000	205,87	1,993	137,26	2,989	103,28	3,972

459	2000	155,63	1,000	78,16	1,991	52,45	2,967	39,48	3,942
	4000	594,14	1,000	298,50	1,990	198,57	2,992	149,54	3,973
	6000	1332,83	1,000	667,31	1,997	444,92	2,996	333,59	3,995

608	800	50,22	1,000	25,66	1,957	17,40	2,886	12,84	3,911
	1200	106,84	1,000	53,91	1,982	35,77	2,987	27,41	3,898
	1600	191,79	1,000	95,93	1,999	64,01	2,996	48,46	3,958

1103	800	164,78	1,000	83,84	1,965	56,83	2,900	41,95	3,928
	1200	359,07	1,000	179,85	1,997	119,89	2,995	89,90	3,994
	1600	628,85	1,000	314,85	1,997	209,78	2,998	158,70	3,963

Mean computational times (in seconds) of the SW algorithm depending on the number of GPUs used. The algorithm was run on Tesla S1070 in U1 rack case containing four graphics cards. The speedup refers always to the one GPU configuration.

To see if the same applies to the NW algorithm and its semiglobal version, we prepared a simple test. The input sequences were taken from the *Ensembl Databases *- *Release *55 and the set of sequences contained 4000 entries with lengths between 100 and 420 amino acids.

All three algorithms, namely global and semiglobal versions of the NW, and the SW algorithm, were launched using again one, two, three and four GPUs, respectively. Each algorithm was run ten times and the mean values of the computational times as well as the speedups are shown in Table 6. The execution times of the algorithms strongly depend on the number of GPUs and the obtained speedup is almost linear. Note that in our implementation multi-GPU support with load balancing works for each alignment algorithm.

**Table 6:** Performance of the algorithms depending on the number of GPUs used

algorithm	1 GPU		2 GPUs		3 GPUs		4 GPUs	
	
	time	speedup	time	speedup	time	speedup	time	speedup
global NW	158,25	1,000	81,86	1,933	54,19	2,920	40,41	3,916

semiglobal NW	163,16	1,000	84,81	1,924	56,01	2,913	41,69	3,914

SW	182,01	1,000	93,89	1,939	61,96	2,938	46,48	3,916

Mean computational times (in seconds) for 4000 input sequences depending on the number of GPUs used. The algorithms were run on Tesla S1070.

### Number of sequences needed to load the GPU

According to Gustafson's law [38], to gain a good speedup on a parallel system, the problem instances have to be sufficiently large. To investigate how large the problem instances must be, the following test was designed. Each dynamic programming algorithm was launched for different problem sizes. The smallest instance consisted of 16 randomly selected sequences from the *Ensembl Databases *- *Release *55 (see Section "Comparison to the Farrar's implementation"). Each subsequent instance contained additional 16 sequences, the largest instance had 1024 sequences. The lengths of the sequences varied between 100 and 420 amino acids. The adopted measure was the average time needed to compute a single alignment of two sequences. To be more precise, the time needed to perform all alignments for a given problem instance was measured and then divided by the total number of computed alignments. The goal was to determine the minimal number of input sequences that could guarantee good performance.

The tests were run on the following hardware:

• CPU: Intel Core 2 Quad Q8200, 2.33 GHz,

• GPU: NVIDIA GeForce GTX 280 with 1 GB of RAM,

• RAM: 8 GB,

• OS: 64-bit Linux.

**Figure 6:**
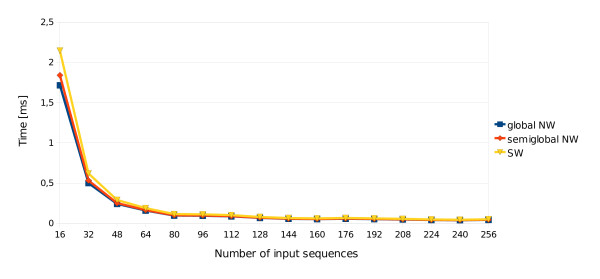
**Average time of computation of one alignment**. Average time of computations of one alignment depending on the total number of input sequences. Tests were run on a single graphics card - GeForce GTX 280.

Figure 6 shows that for sixteen input sequences the average times of performing a single pairwise alignment for the global and semiglobal versions of NW, and the SW algorithm were relatively long - about 1.72, 1.84 and 2.15 milliseconds, respectively. These times were significantly shorter for larger sizes of the problem.

The reasonable times of 0.1, 0.1 and 0.12 ms, respectively, have been achieved for an instance with 80 sequences. The chart is limited to the maximum instance size of 256 sequences, because the curve almost reaches its asymptote. For an instance of this size the times were 0.04, 0.04 and 0.05 ms, respectively. All three algorithms needed only around 0.03 ms if 512 sequences were processed. Although further incrementing of the problem size resulted in some decreases in time, the benefits were not considerable. It means that even for relatively small instances the algorithms were able to gain a good speedup.

### Test on the Fermi architecture

The algorithm was designed during the time when only the G80 and the GT200 GPU architectures were available on the market. However, recently a new architecture, called Fermi [39], has come along. Hence, we set out to check if the application can benefit from a doubled number of CUDA cores that are on the new chips.

The sets of input sequences were the same as in the case of the test from Section "Comparison to the Farrar's implementation". Only the longest sequences were excluded, because of more limited memory on the graphics cards.

The tests were run on the following hardware:

• CPU: Intel Core i7 950, 3.06 GHz,

• GPU: 2× NVIDIA GeForce GTX 480 with 1.5 GB of RAM,

• RAM: 8 GB,

• OS: 64-bit Linux.

Each method, i.e. the SW algorithm as well as the NW algorithm and its semiglobal version, was launched ten times for each input data. Table 7 presents the average performance measured in GCUPS. The standard deviation values *σ *were insignificant (less than 1% of measurement) and hence omitted. Additionally, the table includes the performance of the previous generation architecture - GT200, represented here by one GPU from the Tesla S1070 (see Section "Comparison to the Farrar's implementation").

**Table 7:** Performance of the algorithms depending on the hardware architecture

algorithm	**avg**.	# of	1 GT200 GPU		1 Fermi GPU		2 Fermi GPUs	
	length	sequences	GCUPS	speedup	GCUPS	speedup	GCUPS	speedup
		4000	2,58	1,00	5,13	1,99	10,02	3,88
	51	8000	2,68	1,00	5,21	1,95	10,13	3,78
		12000	2,71	1,00	5,25	1,94	10,28	3,79
	
		2000	2,65	1,00	5,37	2,03	10,55	3,98
	154	4000	2,80	1,00	5,57	1,99	10,87	3,88
SW		6000	2,86	1,00	5,65	1,97	11,07	3,87
	
		2000	2,68	1,00	5,12	1,91	9,91	3,69
	257	4000	2,83	1,00	5,25	1,85	10,21	3,60
		6000	2,90	1,00	5,20	1,80	10,10	3,48
	
		2000	2,71	1,00	4,26	1,57	8,02	2,96
	459	4000	2,84	1,00	4,57	1,61	8,46	2,98
		6000	2,85	1,00	4,64	1,63	8,56	3,01

		4000	3,04	1,00	5,68	1,87	11,13	3,66
	51	8000	3,15	1,00	5,76	1,83	11,21	3,56
		12000	3,17	1,00	5,81	1,83	11,36	3,58
	
		2000	3,10	1,00	5,88	1,90	11,46	3,69
	154	4000	3,29	1,00	6,16	1,87	12,06	3,67
		6000	3,36	1,00	6,28	1,87	12,24	3,64
	
global NW		2000	3,15	1,00	5,68	1,80	10,85	3,44
	257	4000	3,33	1,00	5,80	1,74	11,15	3,35
		6000	3,55	1,00	5,78	1,63	11,15	3,14
	
		2000	3,19	1,00	4,84	1,52	9,07	2,84
	459	4000	3,35	1,00	5,14	1,54	9,63	2,88
		6000	3,36	1,00	5,17	1,54	9,68	2,89

		4000	2,88	1,00	5,50	1,91	10,75	3,74
	51	8000	2,97	1,00	5,58	1,88	10,86	3,66
		12000	3,01	1,00	5,62	1,87	11,04	3,67
	
		2000	3,00	1,00	5,81	1,94	11,36	3,79
	154	4000	3,18	1,00	6,02	1,90	11,87	3,74
		6000	3,25	1,00	6,17	1,90	12,00	3,69
	
semiglobal NW		2000	3,05	1,00	5,60	1,84	10,90	3,57
	257	4000	3,23	1,00	5,77	1,79	11,10	3,44
		6000	3,39	1,00	5,73	1,69	11,03	3,26
	
		2000	3,09	1,00	4,78	1,54	9,04	2,92
	459	4000	3,24	1,00	5,04	1,56	9,55	2,95
		6000	3,25	1,00	5,14	1,58	9,56	2,94

Mean performance (in GCUPS) for different versions of the algorithm running on two architectures: GT200 (Tesla S1070) and Fermi (GeForce GTX480). The columns with speedup always refer to the configuration with one GT200 GPU.

The test shows that with the Fermi architecture the performance of the algorithms increases by a factor of nearly two, especially for short sequences. In case of longer sequences this dominance is slightly reduced, because only 1.5 GB of RAM was available on our Fermi graphics card whereas one Tesla has 4 GB. Obviously, Fermi GPU with 3 or 6 GB of memory may solve this performance issue. However, one should remember that the solution aims to process mainly short and medium-length sequences.

### The backtracking routine overhead

Although the algorithm has been designed especially to deal well with backtracking routine, we also carried out a special test to investigate its performance when the backtracking is not performed, i.e. for score-only version. In other words we set out to check the overhead needed by the backtracking procedure. The *kernel *responsible for the actual backtracking is very quick and as stated before comprises less than 1 percent of total runtime of the algorithm. It, however, requires the backtracking arrays to be filled by the *kernel 1*. Since these arrays are not used for any other purpose, we excluded them from computations.

Tests were conducted on workstation already described in Section "Test on the Fermi architecture". This time, though, only one GPU was used. The sets of input sequences were also the same. The results are presented in Table 8.

**Table 8:** The overhead of the backtracking procedure

avg. length	# of sequences	SW		global NW		semiglobal NW	
		
		GCUPS	BT share	GCUPS	BT share	GCUPS	BT share
51	4000	7,59	1,48	8,35	1,47	8,08	1,47
	8000	7,60	1,46	8,36	1,45	8,31	1,49
	12000	7,61	1,45	8,43	1,45	8,15	1,45

154	2000	8,05	1,50	8,77	1,49	8,89	1,53
	4000	8,24	1,48	9,11	1,48	9,09	1,51
	6000	8,31	1,47	9,17	1,46	9,39	1,52

257	2000	7,76	1,52	8,69	1,53	8,69	1,55
	4000	8,02	1,53	8,65	1,49	8,82	1,53
	6000	7,85	1,51	8,67	1,50	8,65	1,51

459	2000	7,03	1,65	7,84	1,62	7,79	1,63
	4000	7,21	1,58	8,17	1,59	8,01	1,59
	6000	7,19	1,55	8,07	1,56	7,91	1,54

Mean performance (in GCUPS) for different versions of the score-only algorithm - without the backtracking procedure. The *BT share *columns refer to the overhead of corresponding algorithms with backtracking routine. Tests were run on a single GeForce GTX480.

The performance of the score-only algorithm increases considerably reaching up to 9.39 GCUPS. The results are not as good as e.g. in CUDASW++2.0, but one should be aware of the fact that our algorithm was optimized to work well together with the backtracking routine. Moreover, our method has a few assumptions (described earlier) that distinguish it from other implementations but at the same time make it more elaborate.

The *BT share *values were computed in the following way: the computational times of the algorithm with the backtracking routine were divided by the runtimes of corresponding score-only versions of the algorithm. As we can see the overhead of the backtracking is mainly caused by the necessity of filling the *C^up^*, *C^left^*, *B^up ^*and *B^left ^*matrices. However, the additional 45% - 65% is still low in comparison to the method proposed by Myers et al. [19]. Their approach requires twice as much computations and hence the overhead is around 100%. Moreover, we expect that the overhead on GPU would be even higher because of multiple reads of entire input sequences from the global memory. We may conclude that for short and medium-length sequences our method appears to be more suitable.

## Conclusions

Although a few GPU-based implementations of the SW algorithm are already known [13-16] most of them address the problem of database scanning and computing only the alignment scores. Our algorithm is able to compute scores and pairwise alignments. Apart from the SW algorithm, we have also implemented global and semiglobal versions of the NW algorithm. Performed tests show that the efficiency of the implementation is excellent. The algorithm is able to align sequences in roughly the same time as the Farrar's solution needs to compute only scores. Yet, its real dominance reveals while short sequences are processed with no performance loss. Moreover, the speed of our GPU-based algorithms can be almost linearly increased when using more than one graphics card. We have also checked what is a minimum reasonable number of input sequences. Performed tests show, that even for about 80 sequences our algorithms are able to gain a good speedup. What is worth noting, all the tests were performed using real sequences.

The NW and SW algorithms with a backtracking routine may have a lot of applications. They can be used as a robust method for multi-pairwise sequence comparisons performed during the first step in all of the multiple sequence alignment methods based on the progressive approach. Another area of interest can be the usage of a GPU-based semiglobal alignment procedure as a part of the algorithm for the DNA assembly problem, being one of the most challenging problem in current biological studies. It has already been shown that in this case a parallel solution can be successfully applied [20]. Using GPU-based approaches we expect that its execution time would be even shorter, because the large number of short sequences is perfectly in line with the benefits of our algorithm.

## Availability and requirements

• Project name: gpu-pairAlign

• Project home page: http://gpualign.cs.put.poznan.pl

• Operating system: Linux

• Programming language: C/C++

• Other requirements: CUDA 2.0 or higher, CUDA compliant GPU, make, g++

• License: GNU GPLv3

• Any restrictions to use by non-academics: none

## Abbreviations

NW: the Needleman-Wunsch algorithm; SW: the Smith-Waterman algorithm; CPU: central processing unit; GPU: graphics processing unit; GPGPU: general-purpose computing on graphics processing units; CUDA: Compute Unified Device Architecture; RAM: random access memory; OS: operating system.

## Authors' contributions

WF, MK and PW conceived of the study and participated in its design. WF proposed the idea of backtracking arrays. WF and MK contributed equally to algorithm design and implementation. MK carried out computational tests. JB and EP participated in the coordination of the project. All authors were involved in writing the manuscript and all of them read and approved its final version.
